# Patientensicherheit in der Rheumatherapie: Labormonitoring bei Methotrexat

**DOI:** 10.1007/s00393-021-00976-7

**Published:** 2021-03-11

**Authors:** Elizabeth Sierocinski, Aniela Angelow, Armin Mainz, Jochen Walker, Jean-François Chenot

**Affiliations:** 1 Abteilung Allgemeinmedizin, Institut für Community Medicine, Universitätsmedizin Greifswald, Fleischmannstr. 6, 17485 Greifswald, Deutschland; 2 Hausarztpraxis Korbach, Korbach, Deutschland; 3 InGef – Institut für angewandte Gesundheitsforschung Berlin (früher Health Risk Institute), Berlin, Deutschland

**Keywords:** Pharmakovigilanz, Medikamentenmonitoring, Methotrexat, Unerwünschte Arzneimittelwirkungen, Handlungsempfehlungen, Pharmacovigilance, Drug monitoring, Methotrexate, Adverse events, Guidelines

## Abstract

**Hintergrund:**

Methotrexat (MTX) ist das das am häufigsten verordnete krankheitsmodifizierende Antirheumatikum. Ein regelmäßiges Labormonitoring wird empfohlen, um Nebenwirkungen wie Hepatotoxizität und Myelotoxizität sowie MTX-Toxizität-begünstigende Zustände wie eingeschränkte Nierenfunktion früh zu erkennen. Zudem wird eine prophylaktische Folsäuregabe empfohlen. Diese Arbeit untersucht, ob die empfohlene Kontrolluntersuchungen und Folsäureverordnungen während der MTX-Therapie durchgeführt werden.

**Material und Methoden:**

Abrechnungsdaten der gesetzlichen Krankenkassen vom 01.01.2009 bis 31.12.2013 wurden analysiert. Aus der Forschungsdatenbank des InGef (Institut für angewandte Gesundheitsforschung Berlin, früher Health Risk Institute) wurden 40.087 Erwachsene mit einer kodierten rheumatischen Erkrankung (ICD-10-Codes M05–M18), ohne Karzinomdiagnose und ohne MTX-Verordnung ≥ 12 Monate vor Erstverordnung extrahiert. Es wurde analysiert, ob Laborkontrollen entsprechend den Handlungsempfehlungen, eine jährliche rheumatologische Betreuung sowie die Verordnung von Folsäure erfolgten.

**Ergebnisse:**

Es begannen 12.451 Patienten eine neue MTX-Therapie im Beobachtungszeitraum. Das Blutbild, die Leberwerte und die Nierenfunktion wurden bei 42–46 % und der Urinstatus bei 14 % der Patienten wie empfohlen untersucht; 84 % befanden sich in regelmäßiger rheumatologischer Betreuung, und 74 % bekamen eine Folsäureprophylaxe. Möglicherweise MTX-assoziierte schwerwiegende Komplikationen wurden in 0,7 bis 3,5 Fällen/1000 Personenjahre beobachtet.

**Diskussion:**

Kontrolluntersuchungen bei MTX-Therapie werden seltener als empfohlen durchgeführt. Möglicherweise MTX-assoziierte Komplikationen sind aus der Praxisperspektive sehr selten. Einerseits sind Maßnahmen für die bessere Koordination der Kontrolluntersuchungen erforderlich. Andererseits müssen der Nutzen des Monitorings und die Abstände der Monitoringintervalle durch empirische Untersuchungen besser belegt werden.

**Zusatzmaterial online:**

Die Online-Version dieses Beitrags (10.1007/s00393-021-00976-7) enthält eine Übersicht über die GOP-Ziffern und deren Bedeutung. Beitrag und Zusatzmaterial stehen Ihnen auf www.springermedizin.de zur Verfügung. Bitte geben Sie dort den Beitragstitel in die Suche ein, das Zusatzmaterial finden Sie beim Beitrag unter „Ergänzende Inhalte“.

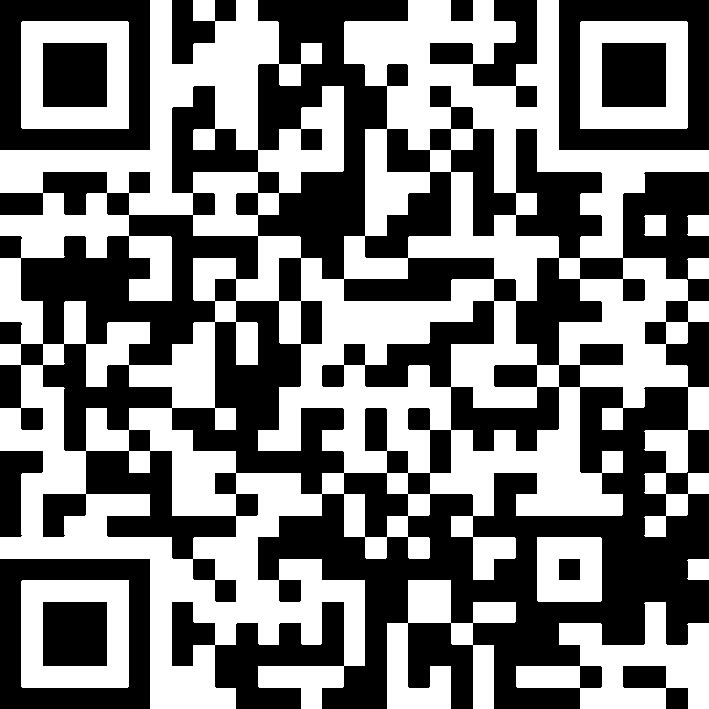

## Abstract (en)

**Background:**

Methotrexate (MTX) is the most commonly prescribed disease-modifying drug in the treatment of rheumatic diseases. Regular laboratory testing is recommended to recognize side effects, such as hepatotoxicity and myelotoxicity as well as decreases in renal function that may cause toxic MTX accumulation. Additionally, folic acid is recommended as prophylaxis against specific side effects. In this study we investigated whether laboratory monitoring and prescription of folic acid took place according to published recommendations.

**Material and methods:**

Claims data from the statutory health insurance from 1 January 2009 to 31 December 2013 were retrospectively analyzed. A total of 40,087 adults with a rheumatic diagnosis (ICD10 codes M05–M18), no malignant disease and no previous MTX prescription within 12 months were extracted from the InGef (Institute for Applied Health Research in Berlin, formerly Health Risk Institute) research database. The frequency of recommended laboratory testing, appointments with rheumatologists and the prescription of folic acid prophylaxis were investigated.

**Results:**

Of the patients 12,451 began treatment with MTX in the observation period. Between 42% and 46% of recommended blood counts, liver values and kidney function tests and 14% of urinalyses were performed according to recommendations. Of the patients 84% were seen regularly by a rheumatologist and 74% received a prescription for prophylactic folic acid. Serious conditions potentially resulting from MTX treatment were observed in 0.7–3.5 cases/1000 person years.

**Discussion:**

Laboratory monitoring in the context of MTX treatment is carried out less frequently than recommended in the literature. Potential MTX-associated serious complications are rare from a practice perspective. On the one hand solutions are needed for a better coordination of laboratory monitoring. On the other hand more empirical evidence is needed regarding the benefits of laboratory monitoring and the appropriate intervals thereof.

Methotrexat (MTX) wird in der Behandlung rheumatologischer Erkrankungen häufig verwendet. Aufgrund der Möglichkeit zahlreicher Nebenwirkungen, darunter Hepatotoxizität und die potenziell tödliche Myelotoxizität, wird ein regelmäßiges Labormonitoring empfohlen. Zudem wird eine prophylaktische Folsäuregabe empfohlen. Diese Arbeit untersucht, ob die empfohlenen Laborkontrollen und Folsäureverordnungen während der MTX-Therapie nach Empfehlungen stattfinden.

## Hintergrund und Fragestellung

Methotrexat (MTX) ist ein krankheitsmodifizierendes Antirheumatikum (DMARD, „disease-modifying antirheumatic drug“) und in Kombination mit einer kurzfristigen Glukokortikoidtherapie Mittel der ersten Wahl in der Behandlung der rheumatoiden Arthritis (RA) [1]. MTX wird von allen synthetischen DMARDs am häufigsten verordnet [2].

Etwa drei Viertel aller MTX-therapierten RA-Patienten erleiden unerwünschte Arzneimittelwirkungen (UAW) [3]. Dies führt in 10–37 % der Fälle zu einem dauerhaften Therapieabbruch [3–5]. UAW wie gastrointestinale und Lebertoxizität sind zwar häufig, aber meist nicht lebensgefährlich. Myelotoxizität sowie die seltene Pneumonitis können lang anhaltende Schädigungen, Behinderungen oder sogar einen vorzeitigen Tod herbeiführen [3, 6–9]. Die Myelotoxizität zeigt sich als besonders gefährlich, indem sie den häufigsten Grund für eine Krankenhauseinweisung zur Behandlung einer MTX-Toxizität (78,5 %) darstellt und eine hohe Mortalitätsrate von 25 % mit sich bringt [7]. Ursächlich sind dabei meist akzidentelle Überdosierungen oder toxische Akkumulationen z. B. bei Nierenversagen. Die Folsäuregabe einen Tag nach der MTX-Gabe verringert zwar gastrointestinale und lebertoxische UAW sowie Therapieabbrüche, aber eliminiert diese nicht vollständig [10].

Um die Zeichen einer MTX-Toxizität möglichst früh zu erkennen und Komplikationen vorzubeugen, empfehlen Experten aus deutschen sowie internationalen Fachgesellschaften ein Monitoring durch regelmäßige Kontrollen des Blutbildes, der Leberwerte und der Nierenretentionsparameter [11–17]. Die Verordnung von Folsäure und eine regelmäßige Betreuung durch einen Facharzt für Rheumatologie werden ebenfalls empfohlen.

Ziel unserer Studie war, durch eine retrospektive Analyse, basierend auf Abrechnungsdaten der gesetzlichen Krankenkasse, zu prüfen, ob das MTX-Monitoring gemäß den Handlungsempfehlungen von der Deutschen Gesellschaft für Rheumatologie (DGRh) und der Deutschen Gesellschaft für Allgemeinmedizin (DEGAM) durchgeführt wird [11, 12]. Zusätzlich wurde die Häufigkeit der Kodierung von potenziell MTX-assoziierten Komplikationen aplastische Anämie und Leberversagen sowie vom Toxizität-begünstigenden akuten Nierenversagen untersucht.

## Studiendesign und Untersuchungsmethoden

### Stichprobe

**Figure 1:**
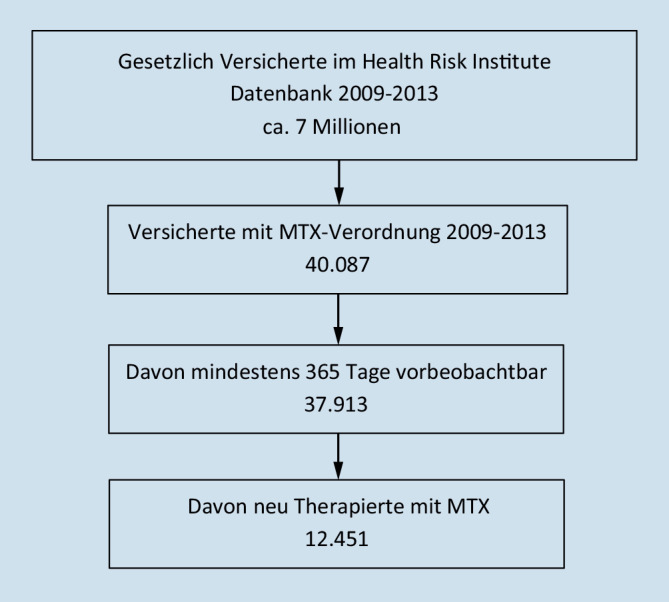

Die retrospektive Analyse basierte auf anonymisierten Abrechnungsdaten der gesetzlichen Krankenkassen. Aus der Forschungsdatenbank des InGef (früher Health Risk Institute, ca. 7 Mio. Versicherte) wurden Erwachsene mit mindestens 2 MTX-Verordnungen (ATC-Code L01BA01) im Zeitraum von 01.01.2009 bis 31.12.2013 extrahiert [18]. Die Nutzung der Datenbank erfolgte gemäß § 284 SGB V (Sozialdaten bei den Krankenkassen) in Verbindung mit § 70 SGB V (Qualität, Humanität und Wirtschaftlichkeit). Eingeschlossen in die Analyse wurden erwachsene Patienten mit einer kodierten rheumatischen Erkrankung (ICD-10-GM M05–M18; Arthritiden und entzündliche Polyarthropathien), keiner Karzinomdiagnose (ICD-10-GM C00–C75) und keiner MTX-Verordnung ≥ 12 Monate vor der Erstverordnung (Neuverordnung) (Abb. 1). Daten wurden bis zu einer MTX-Therapielücke von ≥ 90 Tagen nach Ende der Verordnungsreichweite erhoben.

### Monitoringparameter

Unter der Annahme, dass das Monitoring zumindest zum Teil durch Hausärzte durchgeführt wird, wurden die Gebührenordnungspositionen (GOPs, Anhang 1, s. elektronisches Zusatzmaterial online) für die Bestimmung der von der Deutschen Gesellschaft für Allgemeinmedizin (DEGAM) empfohlenen Laboruntersuchungen genutzt (diese Empfehlungen stimmten weitgehend mit denen der Deutschen Gesellschaft für Rheumatologie [DGRh] überein; Tab. 1). Die Häufigkeit der rheumatologischen Betreuung wurde ebenfalls mittels GOPs untersucht. Mittels ATC-Code (B03BB) wurde die von der DGRh empfohlene Verordnung für prophylaktische Folsäure gemäß Anlage 1 der Arzneimittelrichtlinie untersucht [19]. Zusätzlich wurden die ICD-10-GM-Codes für Ereignisse bestimmt, die potenziell auf MTX zurückzuführen sind (aplastische Anämie ICD D61.1*, Leberversagen ICD K71.1) oder die MTX-Toxizität begünstigen könnten (akutes Nierenversagen ICD N17.*).

**Table 1:** 

Gesellschaft	Maßnahmen	Häufigkeit
*Deutsche Gesellschaft für Rheumatologie e.* *V. (DGRh)*^*a*^	Blutbild einschließlich Thrombozyten und Differenzialblutbild, GOT, GPT, alkalische Phosphatase, Kreatinin	Vor Therapiebeginn Nach 1 bis 2 Wochen Dann nach weiteren 2 bis 3 Wochen Danach alle 4 Wochen
	Befragung und klinische Untersuchung: Exanthem, Stomatitis, gastrointestinale Symptome, Fieber, Luftnot, Husten, Blutungen	Bei längerer komplikationsloser Verträglichkeit weitere Streckung auf 6 bis 12 Wochen möglich^b^
	Folsäure 5 mg	Einen Tag nach der MTX-Gabe
*Deutsche Gesellschaft für Allgemeinmedizin und Familienmedizin (DEGAM)*	BB, GGT, GPT, Kreatinin, Urinstatus	Im ersten Monat: wöchentlich Im 2. und 3. Monat: alle 2 Wochen Ab dem 4. Monat: 1‑mal im Quartal

*BB* Blutbild, *GGT* Gamma-Glutamyltransferase, *GPT* Glutamat-Pyruvat-Transaminase, *eGFR* Kreatinin (Nierenfunktion), Urinstatus: Diagnostik mittels Urinteststreifen

^a^Die Empfehlungen sind fast identisch mit Expertenempfehlungen aus 2009 (Tarner et al. 2009) [13]

^b^Beibehalten der engmaschigen Kontrolle wird bei älteren Patienten mit chronischen Erkrankungen empfohlen

### Analysen

Die Abrechnungsdaten wurden deskriptiv ausgewertet. Anhand der Häufigkeit der Kodierungen für die empfohlenen Maßnahmen wurde analysiert, welcher Patientenanteil über die gesamte Therapiedauer gemäß der DEGAM-S1-Leitlinie (Handlungsempfehlungen nach Klassifikation der Arbeitsgemeinschaft der wissenschaftlichen medizinischen Fachgesellschaften [AWMF]) untersucht wurde. Die Angaben beziehen sich immer auf die zu dem Zeitpunkt beobachtbaren Patienten. Die Häufigkeit der Komplikationen wurde durch Inzidenzraten von Fällen pro 1000 Personenjahre berechnet. Die Auswertungen erfolgten mit R 3.2.1.

## Ergebnisse

### MTX-Verordnungen

Insgesamt 12.451 Patienten begannen eine neue MTX-Therapie zwischen 2009 und 2013. Die Zahl der beobachtbaren Patienten nahm in jedem Quartal ab. Nach einem Jahr erhielt nur noch ca. die Hälfte (*n* = 5914) eine MTX-Verordnung. Das mittlere Alter der Patienten betrug 53,8 Jahre (±SD 14,1), und 64 % waren Frauen (Tab. 2). Die mittlere Therapiedauer betrug 476 Tage.

**Table 2:** 

*Mittleres Alter in Jahren (SD)*	53,8 (±14,1)
*Minimum Alter*	18
*Maximum Alter*	95
*Anteil Frauen*	64,0 %

### Labormonitoring

**Figure 2:**
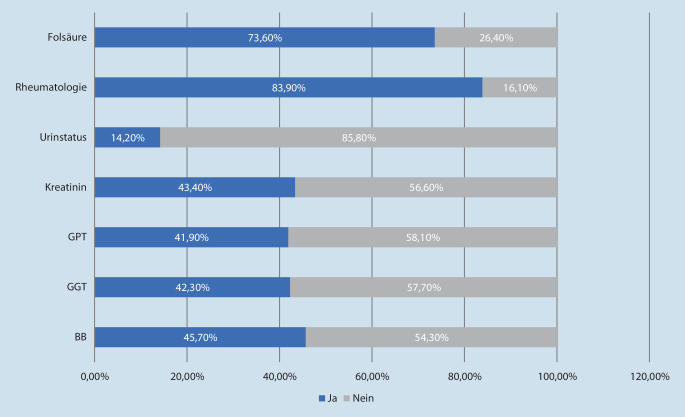

Die von der DEGAM empfohlenen Laborkontrollen (im ersten Behandlungsmonat wöchentlich, in den 2. und 3. Monaten alle 2 Wochen und danach 1‑mal in Quartal) wurden während der gesamten Therapiedauer bei 46 % der Patienten für das Blutbild, 42 % für die GGT, 42 % für die GPT, 43 % für das Kreatinin und 14 % für den Urinstatus abgerechnet (Abb. 2). Eine rheumatologische Betreuung wurde in 84 % der Fälle jährlich abgerechnet. Die von der DGRh empfohlene Folsäureverordnung lag bei 74 % der Patienten vor.

**Figure 3:**
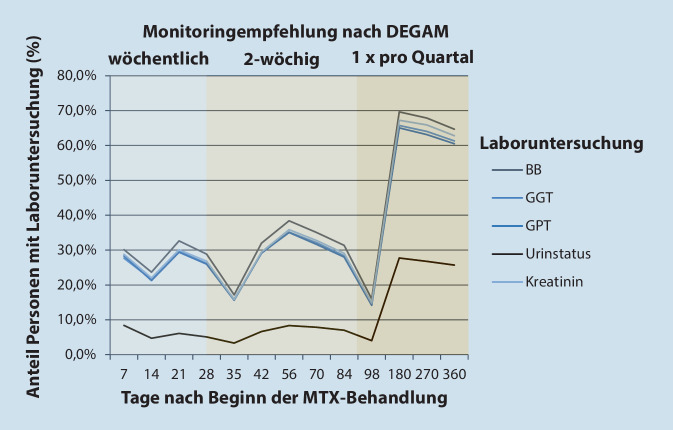

Die Durchführung der empfohlenen Laborkontrollen erfolgte seltener als empfohlen insbesondere zu Beginn der Therapie mit einer Bestimmung von Blutbild in 30,1 % der Patienten, von der GGT in 27,7 %, von der GPT in 28,3 %, von Kreatinin in 28,8 % und Urinstatus in 8,4 % in den ersten 7 Therapietagen. In dem Zeitraum zwischen dem 91. und 180. Therapietag betrug der untersuchte Patientenanteil 69,6 % für das Blutbild, 65,7 % für die GGT, 65,0 % für die GPT, 67,2 % für Kreatinin und 27,7 % für den Urinstatus (Abb. 3).

### Potenzielle Komplikationen und UAW-begünstigende Zustände

Akutes Nierenversagen wurde in 3,5 Fällen/1000 Personenjahre (0,3 %), Leberversagen in 0,7 Fällen/1000 Personenjahre (0,1 %) und aplastische Anämie in 0,9 Fällen/1000 Personenjahre (0,1 %) in den Abrechnungsdaten kodiert (Tab. 3).

**Table 3:** 

Komplikation	Fälle/1000 Personenjahre	95 %-CI
Akutes Nierenversagen	3,48	2,64–4,51
Leberversagen	0,68	0,34–1,22
Aplastische Anämie	0,93	0,52–1,53

## Diskussion

### Zusammenfassung

Die Kontrolluntersuchungen bei MTX-Therapie wurden seltener durchgeführt, als in der DEGAM-Leitlinie empfohlen. Abweichungen von den Empfehlungen zeigten sich insbesondere zu Beginn der Therapie. Bei knapp drei Viertel der Patienten wurde Folsäure verordnet, und mehr als 80 % der Patienten befanden sich in rheumatologischer Betreuung. Potenziell auf MTX zurückführbare Komplikationen und Toxizität-begünstigendes akutes Nierenversagen konnten beobachtet werden.

### Notwendigkeit des Monitorings

Unsere Daten weisen auf ein selteneres Monitoring als in den Empfehlungen der DEGAM, der DGRh und internationaler Gesellschaften angegeben. Betont in diesen Empfehlungen werden mindestens 4‑wöchentliche Laborkontrollen am Anfang der MTX-Therapie [11, 12], da UAW wie Leberschädigungen und Myelotoxizität häufiger in den ersten Monaten der Therapie auftreten [3]. Nur bei längerer komplikationsloser Verträglichkeit der MTX-Therapie sollten seltenere Kontrollen alle 6 bis 12 Wochen in Erwägung gezogen werden [11, 12, 14–17]. Es besteht ein Risiko für UAW für die gesamte Therapiedauer [8]: Leberwerterhöhungen bis hin zu schwerer Lebertoxizität treten in bis zu einem Drittel der MTX-Therapierten auf [5, 20]. Die seltene (5 %), aber tödliche (25 % Mortalitätsrate bei hospitalisierten Patienten) Myelotoxizität kann durch einen erhöhten MTX-Spiegel aufgrund nur leichter, passagerer Einschränkung der Nierenfunktion, bei Interaktionen mit anderen Medikamenten oder Fehldosierungen (z. B. versehentliche tägliche, nicht wöchentliche Gabe) auftreten [5, 6]. In unserer Studie beobachteten wir Ereignisse, die potenzielle Komplikationen einer MTX-Therapie darstellten (Leberversagen und aplastische Anämie) oder eine toxische Erhöhung des MTX-Spiegels verursachen konnten (akutes Nierenversagen). Das Auftreten dieser Ereignisse, bei denen eine Implikation von MTX erwogen wird, kann eine Begründung für ein langfristiges Monitoring sein. Allerdings gibt es keine empirische Datengrundlage für optimale Monitoringintervalle. Die Empfehlungen basieren lediglich auf Expertenkonsens.

Eine französische Studie zeigt ebenfalls ein selteneres MTX-Monitoring, als in Handlungsempfehlungen dargestellt [21]. In dieser Studie werden häufigere Kontrolluntersuchungen mit früheren MTX-Therapieabbrüchen assoziiert [21]. Ob diese Therapieabbrüche UAW oder deren Komplikationen mindern, ist unklar. Es gibt keine Evidenz, ob die oft akut auftretenden schwerwiegenden Komplikationen überhaupt durch das Monitoring vermieden oder reduziert werden können oder ob Labormonitorings im Allgemeinen eher unnötige Eingriffe und Arbeitsaufwände für den Patienten und Arzt darstellen [22].

### Zugrunde liegende Faktoren

Die Diskrepanz zwischen Handlungsempfehlungen und der Durchführung des Monitorings wurde bisher in der Literatur nicht geklärt. Fehlende Informationen, Zeit- und Ressourcenmangel sowie Unterversorgung stellen mögliche Einflussfaktoren dar. Ein hoher Anteil der Patienten in unserer Studie wurde rheumatologisch betreut. Die verfügbaren Daten erlauben es nicht, die Absprachen oder mangelnden Absprachen zur Koordination einzelner Versorgungsaufgaben zwischen Hausärzten und Rheumatologen nachzuvollziehen. Es ist möglich, dass Patienten nicht über die Notwendigkeit des Monitorings und Hausärzte über ihre Rolle als Koordinatoren des Monitorings von den MTX verordnenden Ärzten informiert werden. In Praxen fehlen möglicherweise auch die notwendige Zeit und Ressourcen, um Patienten regelmäßig für das Monitoring einzubestellen. Es ist möglich, dass mit MTX behandelte Patienten im Rahmen des Ärztemangels in Deutschland hausärztlich und rheumatologisch unterversorgt sind und deswegen keine Monitoringtermine wahrnehmen. Weiterhin könnte eine mangelnde Adhärenz unabhängig von der Versorgungslage eine Rolle spielen.

### Potenzielle Lösungen

Eine Methode, um Monitoringuntersuchungen in der Versorgung chronischer Erkrankungen zu etablieren, sind Disease-Management-Programme (DMP), weil diese mit einer extrabudgetären Vergütung verknüpft sind. Das in 2005 und 2014 beim Gemeinsamen Bundesausschuss (G-BA) beantragte DMP bei Rheuma ist zugunsten anderer Volkserkrankungen verschoben worden [23]. Eine Verbesserung der direkten Kommunikation zwischen Spezialisten und Hausärzten ist aufgrund des bereits zeitlich angespannten Praxisalltags nur eingeschränkt durchführbar. Jedoch muss die Zuständigkeit für die Kontrollen zwischen Rheumatologen und Hausärzten besser abgesprochen und koordiniert werden. Zum Beheben des Informationsdefizits könnten aktuelle Handlungsempfehlungen an Hausarztpraxen disseminiert werden. Um das Monitoring zu begünstigen und Patienten regelmäßig einzubestellen, kämen Softwarelösungen in der elektronischen Patientenakte infrage, die ein Kontrollschema beinhalten und Fachangestellte über notwendige Monitoringtermine informieren. Patienten können über die Notwendigkeit des Monitorings breitflächig in einem standardisierten Aufklärungsverfahren mittels eines Aufklärungsvideos informiert werden.

### Standardisierung der Empfehlungen

Die verfügbaren Empfehlungen stimmen in den zeitlichen Abständen von Monitoringuntersuchungen großenteils überein, aber die empfohlenen Kontrollparameter und Maßnahmen unterscheiden sich. Beispielsweise wird die Erhebung eines Urinstatus in der DEGAM-Leitlinie und in der MTX-Fachinformation [24] empfohlen, aber weder von der Deutschen Gesellschaft für Rheumatologie noch von anderen internationalen Gesellschaften erwähnt [11, 12]. Dies gilt als mögliche Erklärung für die besonders niedrige Anzahl an durchgeführten Urinanalysen in unserer Studie. Andere Handlungsempfehlungen empfehlen eine Thoraxröntgenaufnahme vor Therapiebeginn, um mögliche Lungenvorerkrankungen und dabei Patienten mit erhöhtem Risiko für pulmonale UAW zu erkennen [17]. Die Notwendigkeit aller empfohlenen Untersuchungen muss überprüft werden, um unnötige Untersuchungen zu vermeiden [25]. Standardisierte Empfehlungen würden Verwirrung und Fehlinformationen minimieren und stellen eine Voraussetzung für die breitflächige Implementierung der oben genannten Lösungsstrategien dar. Eine wichtige Voraussetzung für standardisierte Monitoringempfehlungen stellt die Verbesserung der Evidenzlage bezüglich der Laborparameter und Monitoringintervalle dar. Dies stellt eine Herausforderung nicht nur für das MTX-Monitoring, sondern für das Labormonitoring chronischer Erkrankungen wie Diabetes und Bluthochdruck dar [22].

### Stärken und Limitationen

Basierend auf den Abrechnungsdaten kann nicht evaluiert werden, ob die Laborbestimmungen direkt auf die MTX-Therapie zurückzuführen sind. Bei den erfassten Ereignissen von Leberversagen, Nierenversagen und aplastischer Anämie mit Krankenhausaufenthalt kann von einer Vollständigkeit ausgegangen werden, allerdings muss MTX nicht kausal verantwortlich sein. Andere Aspekte des Medikamentenmonitorings wie Therapietreue, Erfassen der Verträglichkeit und Wirksamkeit auf Krankheitsaktivität können mit Abrechnungsdaten nicht abgebildet werden. Dies stellt laut DGRh-Therapieüberwachungsbogen einen unerlässlichen Teil des Monitorings dar [12]. Es ist möglich, dass Laborkontrollen und insbesondere Urinstatus nicht kodiert worden sind oder mit anderen als die in unserer Studie benutzten EBM-Ziffern abgerechnet worden sind. Es ist möglich, dass wir den Anteil der Patienten, die Folsäure erhalten, unterschätzen, da dies auch frei verkäuflich ist. Die Zahl der beobachtbaren Patienten nahm im Beobachtungszeitraum ständig ab. Die Gründe dafür lassen sich aus Abrechnungsdaten nicht nachvollziehen.

## Fazit für die Praxis

Die Verordnung von MTX steigt jährlich.

Das Labormonitoring wird deutlich seltener durchgeführt, als in Handlungsempfehlungen empfohlen wird.

Weitere Studien sollen die zugrunde liegenden Faktoren ermitteln; eine Evidenzlage für standardisierte Empfehlungen ist ebenfalls notwendig.

Einerseits sind Maßnahmen für die bessere Koordination der Kontrolluntersuchungen, wie z. B. Softwarelösungen oder Aufklärungsvideos für die Durchführung eines empfohlenen MTX-Labormonitorings, erforderlich, andererseits müssen der Nutzen des Monitorings und die Abstände der Monitoringintervalle durch empirische Untersuchungen besser belegt werden.

## Supplementary Information



## Abkürzungen

ATC: Anatomisch-therapeutisch-chemisches Klassifikationssystem
EBM: Einheitlicher Bewertungsmaßstab
GKV: Gesetzliche Krankenversicherung
GOP: Gebührenordnungsposition
ICD: Internationale statistische Klassifikation der Krankheiten und verwandter Gesundheitsprobleme
