# Supplementary material for: Patient safety in the treatment of rheumatic diseases: Laboratory monitoring in methotrexate treatment
Source: Z Rheumatol. 2021 Mar 11;80(5):418–24. [Article in German] doi: 10.1007/s00393-021-00976-7 (PMC8189943; doi:10.1007/s00393-021-00976-7)
Supplement: Supplementary file 1 [file 393_2021_976_MOESM1_ESM.pdf]

Anhang 1. GOP-Ziffern und deren Bedeutung

Name	Ziffer	Bedeutung
Rheuma	04550	Zusatzpauschale
Rheuma	04551	Zusatzpauschale spezielle Funktionsdiagnostik in der pädiatrischen Rheumatologie
Rheuma	13690	Rheumatologische Grundpauschale bis zum vollendeten 5. Lebensjahr
Rheuma	13691	Rheumatologische Grundpauschale zwischen dem 6.- 59. Lebensjahr
Rheuma	13692	Rheumatologische Grundpauschale ab dem 60. Lebensjahr
Rheuma	13700	Zusatzpauschale internistische Rheumatologie
Rheuma	13701	Zusatzpauschale rheumatologische Funktionsdiagnostik
Rheuma	18320	Zusatzpauschale orthopädische oder orthopädisch-rheumatologische Funktionsdiagnostik bzw. Assessment mittels Untersuchungsinventaren
Rheuma	18700	Zusatzpauschale Behandlung von rheumatoider Arthritis, seronegativer Spondylarthritis, Kollagenose, Myositis
Rheuma	32023	Rheumatoide Arthritis einschl. Sonderformen und Kollagenosen unter immunsuppressiver oder immunmodulierender Langzeit-Basistherapie
eGFR	32066	Kreatinin (Jaffé-Methode)
eGFR	32067	Kreatinin, enzymatisch
eGFR	17340	Zusatzpauschale Nierenfunktionsdiagnostik
eGFR	32124	Endogene Kreatininclearance
eGFR	32236	Kreatin
GPT	32070	GPT (Glutamat-Pyruvat-Transaminase)
GGT	32071	GGT (Gamma-Glutamyl-Transferase)
BB	32120	Mechanisiertes Blutbild, Retikulozytenzählung und evtl. Erythrozytenzahl, Leukozytenzahl (ggf. einschl. orientierender Differenzierung), Thrombozytenzahl, Hämoglobin, Hämatokrit
BB	32121	Mechanisierte Leukozytendifferenzierung
BB	32122	Mechanisierter vollständiger Blutstatus
UrinStix	32030	Orientierende Untersuchung
UrinStix	32135	Urin-Mikroalbumin
UrinStix	32880	Harnstreifentest
